# Expression of Concern: Effects of microRNA-136 on melanoma cell proliferation, apoptosis and epithelial mesenchymal transition by targeting PMEL through the Wnt signaling pathway

**DOI:** 10.1042/BSR-20170743_EOC

**Published:** 2020-08-28

**Keywords:** Cell proliferation, Epithelial-mesenchymal transition, Melanoma cells, microRNA-136, PMEL, Wnt signaling pathway

The Editorial Office has been made aware of potential issues surrounding the scientific validity of this paper, hence has issued an expression of concern to notify readers whilst the Editorial Office investigates.

